# Supplementary material for: Using symmetry to elucidate the importance of stoichiometry in colloidal crystal assembly
Source: Nat Commun. 2019 May 2;10:2028. doi: 10.1038/s41467-019-10031-4 (PMC6497718; doi:10.1038/s41467-019-10031-4)
Supplement: Supplementary file 1 — Supplementary Information [file 41467_2019_10031_MOESM1_ESM.pdf]

Supplementary Information for "Using symmetry to elucidate the importance of stoichiometry in colloidal crystal assembly"

Nathan A. Mahynski,[1, a] Evan Pretti,[2] Vincent K. Shen,[1] and Jeetain Mittal[2, b]

[1] *Chemical Sciences Division, National Institute of Standards and Technology, Gaithersburg, Maryland 20899-8320, USA*

[2] *Department of Chemical and Biomolecular Engineering, Lehigh University, 111 Research Dr., Bethlehem, Pennsylvania 18015-4791, USA*

[a] Electronic mail: nathan.mahynski@nist.gov

[b] Electronic mail: jeetain@lehigh.edu

SUPPLEMENTARY FIGURES

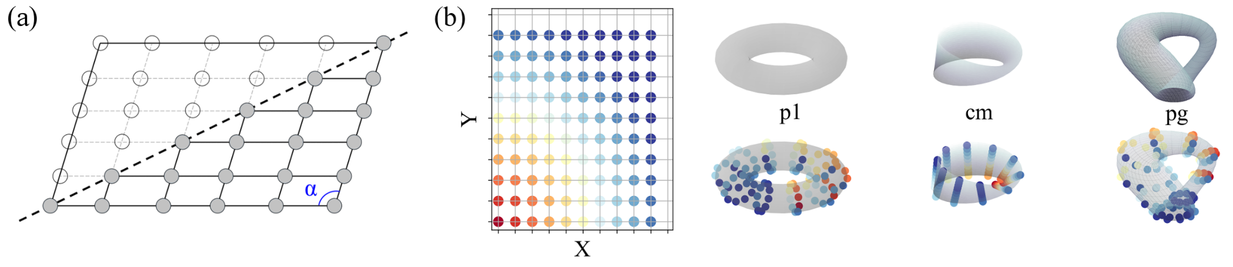

Supplementary Figure 1: Orbifolding the fundamental domain. (a) A general parallelogram used to model fundamental domains is shown covered by a lattice of points (also see Fig. 1 in the main text). (b) A rectangular fundamental domain with colored lattice sites is wrapped according to three different groups' symmetries to produce their respective orbifolds; p1 corresponds to a torus, cm to a Möbius strip, and pg to a Klein bottle.

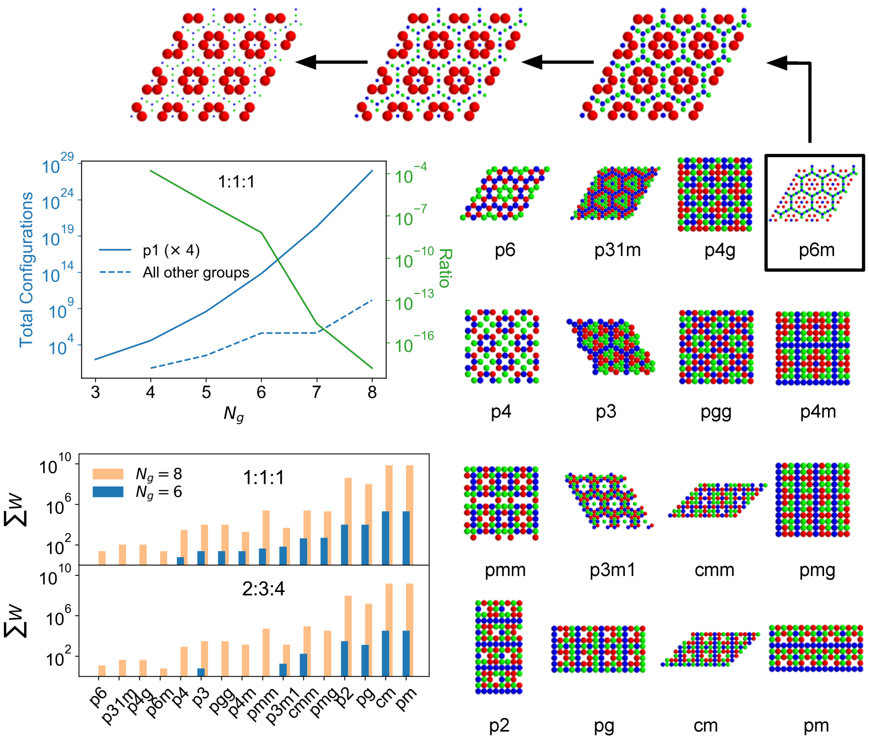

Supplementary Figure 2: Enumeration of solutions to the constraint satisfaction problem (CSP) for ternary systems. The total number of configurations, representing the sum of all realizations of all solutions to the CSP for all of the wallpaper groups besides p1, is given by the dashed blue line for a 1:1:1 mixture at various N_g. When not constrained by the symmetry of a given group, we set the sides of its fundamental domain equal to each other and $\alpha = \pi/2$. The solid blue line is the number of solutions for a total of 4 different p1 cells, each with different angles in the fundamental domains (*cf.* main text); the green line is the ratio between the two blue ones. Randomly chosen configurations for each group are also depicted which have been scaled to contact for equally sized colloids. A breakdown of the number of solutions each group contributes is also provided for representative N_g values and stoichiometries. Above, the p6m group's solution has been scaled to contact assuming different diameters for the red colloids to illustrate how the same pattern can be used for differently sized colloids.

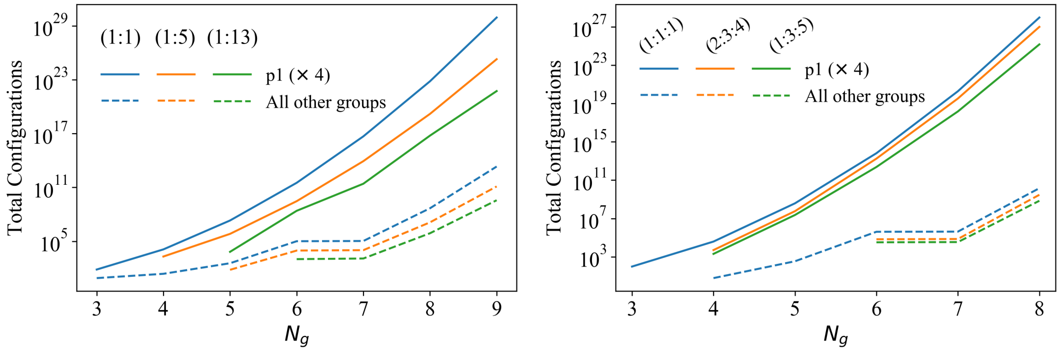

Supplementary Figure 3: Total number of configurations for different N_{g} for binary (left) and ternary (right) mixtures. As in the main text, these are compared against the combinatorial explosion occurring for p1, for which 4 different angles are considered, $\alpha \in [\pi/2, \pi/3, \pi/4, \pi/6]$, hence the multiplication by this factor.

In Supplementary Fig. 3 we report representative results for binary and ternary mixtures. For sufficiently small N_{g} no solutions exist, though this bound depends on the stoichiometry. Both the binary and ternary cases exhibit similar behavior, displaying a combinatorial explosion for p1 which is largely suppressed compared to the sum of configurations resulting from all other groups with congruent primitive cells. A breakdown of this total is provided in Supplementary Figs. 4 and 5 for binary and ternary mixtures, respectively. Generally, the number of configurations is proportional to the number of nodes per edge, N_1, for each group, but not always.

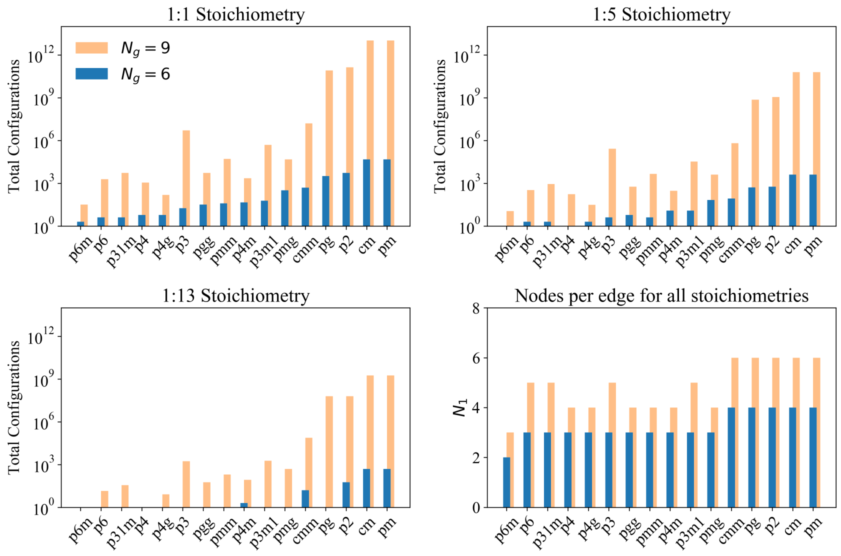

Supplementary Figure 4: Breakdown based on group of the total number of configurations for a binary mixture with different stoichiometries. The number of nodes per edge on the fundamental domain, N_1, resulting from each N_g considered here is also presented.

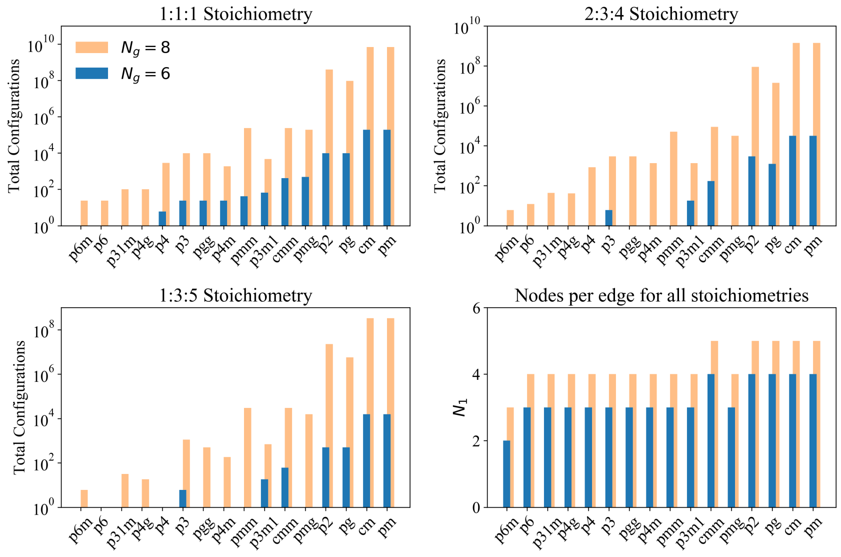

Supplementary Figure 5: Breakdown based on group of the total number of configurations for a ternary mixture with different stoichiometries. The number of nodes per edge on the fundamental domain, N_1, resulting from each N_g considered here is also presented.

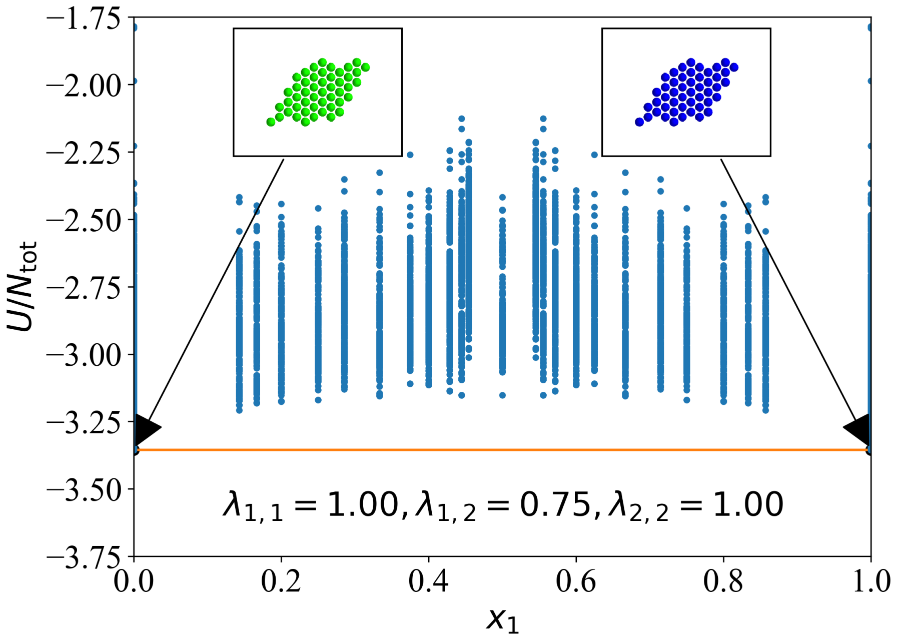

Supplementary Figure 6: Phase diagram for a binary mixture at $(\lambda_{1,1}, \lambda_{1,2}, \lambda_{2,2}) = (1.0, 0.75, 1.0)$. The convex hull of potential energy is drawn in orange, the structure candidates are indicated by blue dots. In this case, only two structures belong to the hull which correspond to hexagonal lattices of each individual component. No energetic degeneracy was found for these lattices on the hull.

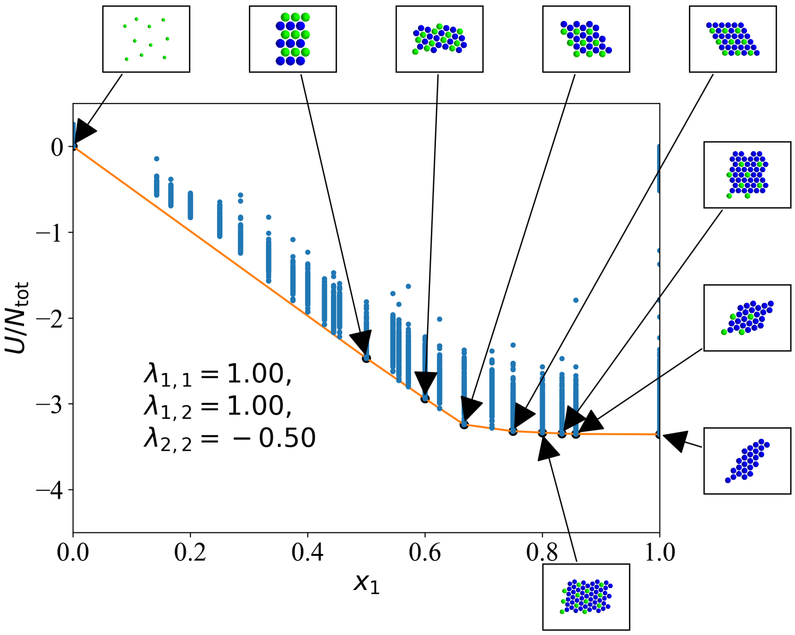

Supplementary Figure 7: Phase diagram for a binary mixture at $(\lambda_{1,1}, \lambda_{1,2}, \lambda_{2,2}) = (1.0, 1.0, -0.5)$. The convex hull of potential energy is drawn in orange, the structure candidates are indicated by blue dots, and all candidates which belong to the hull are indicated with an image. No energetic degeneracy was found for these lattices on the hull.

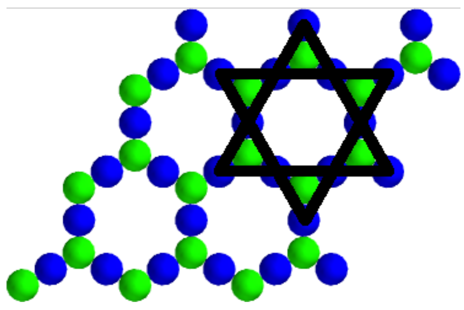

Supplementary Figure 8: This structure may be viewed as a Kagome lattice formed by equilateral triangles passing through the centers of mass of only the majority component (blue), and has a 3:2 stoichiometry.

SUPPLEMENTARY TABLES

Group	α Constraint	L_2/L_1 Constraint	Supercell
p1 (o)	None	None	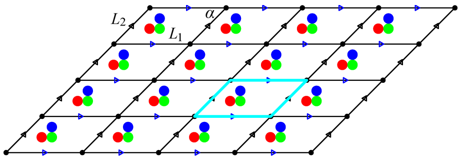
p2 (2222)	None	None	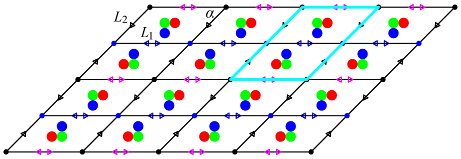
p3 (333)	$\alpha = \pi/3$	1	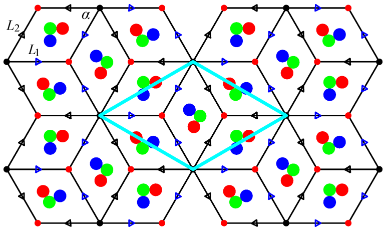
p4 (442)	$\alpha = \pi/2$	1	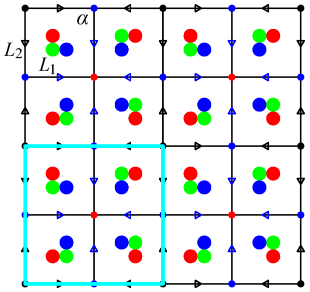
p6 (632)	$\alpha = 2\pi/3$	1	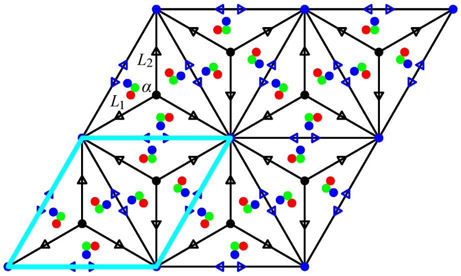
pm (**)	$\alpha = \pi/2$	None	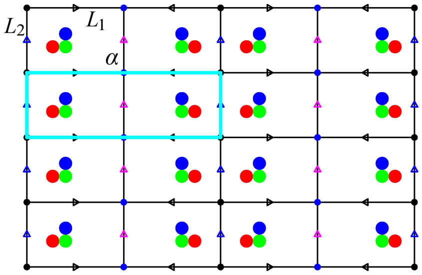

pmm (*2222)	$\alpha = \pi/2$	None	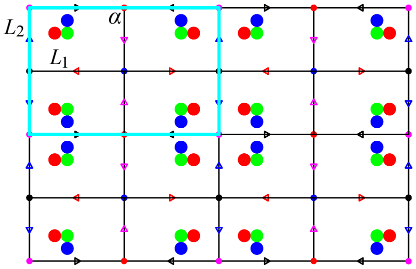
p4m (*442)	$\alpha = \pi/2$	1	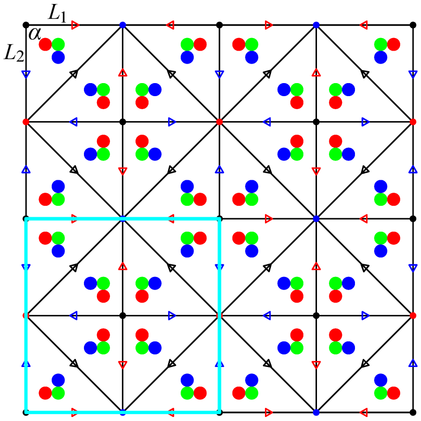
p6m (*632)	$\alpha = \pi/3$	2	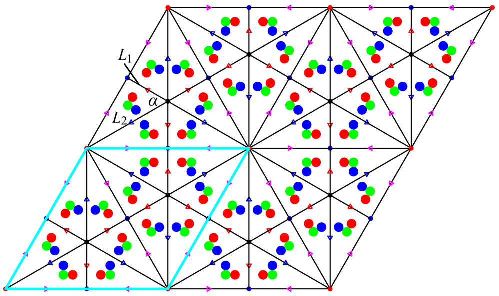
p3m1 (*333)	$\alpha = \pi/3$	1	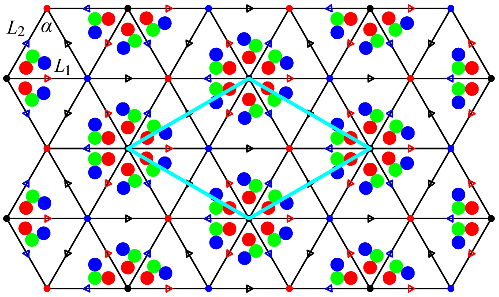
p31m (3*3)	$\alpha = 2\pi/3$	1	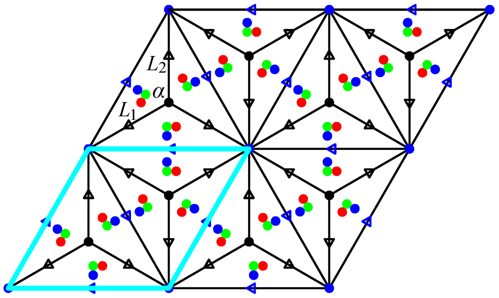

p4g (4*2)	$\alpha = \pi/2$	1	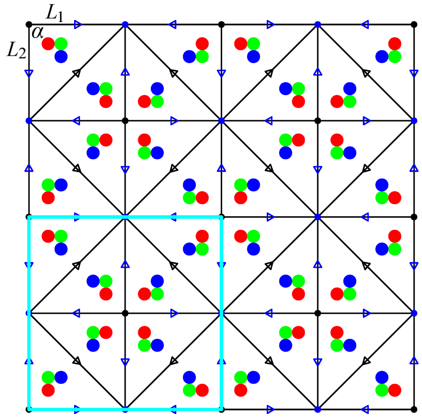
cmm (2*22)	$\alpha = \pi/2$	None	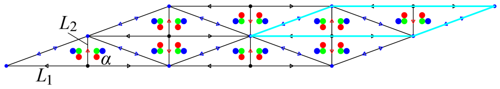
pmg (22*)	$\alpha = \pi/2$	None	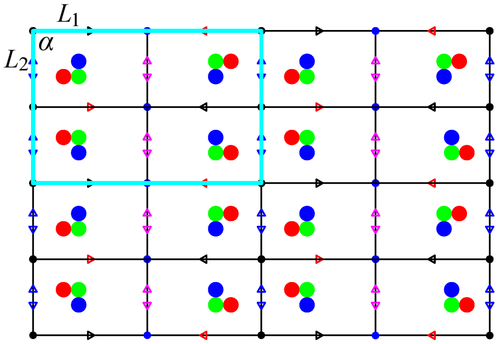
pg (xx)	$\alpha = \pi/2$	None	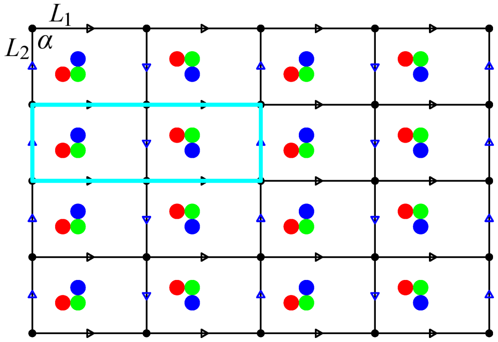
cm (*x)	$\alpha = \pi/2$	None	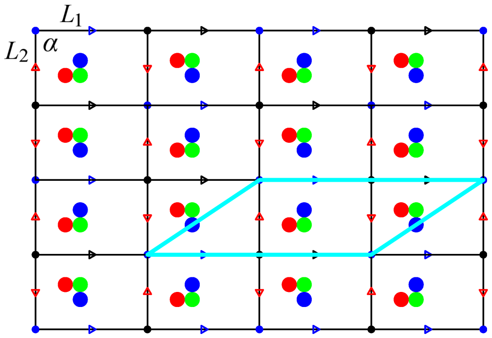

pgg (22x)	$\alpha = \pi/2$	None	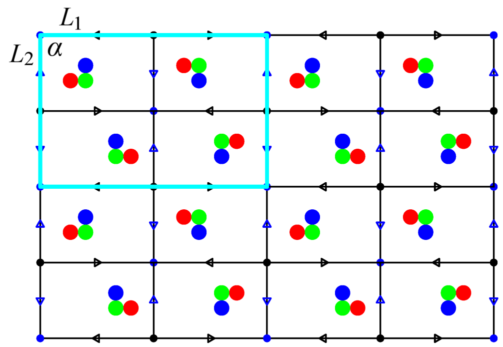

Supplementary Table I: Wallpaper groups used in our algorithm. The short Hermann-Mauguin, or International Union of Crystallography, name for each group is given followed by its orbifold notation in parentheses. The fundamental domain for each group is given by the smallest polygon (either a parallelogram or triangle) bounded by black lines while the primitive cell is indicated by the cyan parallelogram. Several primitive cells are repeated for each group to show how this cell tiles space by translation along its vectors. The chiral RGB-trimer is used to indicate orientation of the fundamental domains relative to each other. Corners are indicated by colored dots; identical corners have the same colored dot. Edges are indicated by colored arrows denoting directionality. As with corners, edges with the same colored arrow are identical in the direction indicated by the relative direction of their arrows. A set of two arrows pointing in opposite directions on the same edge indicates the edge has mirror symmetry centered at its midpoint; *i.e.*, the edge itself is identical moving outward from its center in both directions.

Group	N_s	N_d	α	r	N_1	N_2
p2 (2222)	4	2	$\{\pi/2, \pi/3, \pi/4, \pi/6\}$	$\{1, \sqrt{2}, \sqrt{3}, 2\}$	$\left\lfloor \frac{N_\mathrm{g}}{\sqrt{2r}} \right\rfloor$	$\left\lfloor \frac{N_\mathrm{g}\sqrt{r}}{\sqrt{2}} \right\rfloor$
p3 (333)	4	3	$\pi/3$	1	$\left\lfloor \frac{N_\mathrm{g}}{\sqrt{3r}} \right\rfloor$	$\left\lfloor \frac{N_\mathrm{g}\sqrt{r}}{\sqrt{3}} \right\rfloor$
p4 (442)	4	4	$\pi/2$	1	$\left\lfloor \frac{N_\mathrm{g}}{2\sqrt{r}} \right\rfloor$	$\left\lfloor \frac{N_\mathrm{g}\sqrt{r}}{2} \right\rfloor$
p6 (632)	3	6	$2\pi/3$	1	$\left\lfloor \frac{N_\mathrm{g}}{\sqrt{3r}} \right\rfloor$	$\left\lfloor \frac{N_\mathrm{g}\sqrt{r}}{\sqrt{3}} \right\rfloor$
pm (**)	4	2	$\pi/2$	$\{1, \sqrt{2}, \sqrt{3}, 2\}$	$\left\lfloor \frac{N_\mathrm{g}}{\sqrt{2r}} \right\rfloor$	$\left\lfloor \frac{N_\mathrm{g}\sqrt{r}}{\sqrt{2}} \right\rfloor$
pmm (*2222)	4	4	$\pi/2$	$\{1, \sqrt{2}, \sqrt{3}, 2\}$	$\left\lfloor \frac{N_\mathrm{g}}{2\sqrt{r}} \right\rfloor$	$\left\lfloor \frac{N_\mathrm{g}\sqrt{r}}{2} \right\rfloor$
p4m (*442)	3	8	$\pi/2$	1	$\left\lfloor \frac{N_\mathrm{g}}{2\sqrt{r}} \right\rfloor$	$\left\lfloor \frac{N_\mathrm{g}\sqrt{r}}{2} \right\rfloor$
p6m (*632)	3	12	$\pi/3$	2	$\left\lfloor \frac{N_\mathrm{g}}{\sqrt{6}} \right\rfloor$	$\left\lfloor \frac{N_\mathrm{g}}{\sqrt{6}} \right\rfloor$
p3m1 (*333)	3	6	$\pi/3$	1	$\left\lfloor \frac{N_\mathrm{g}}{\sqrt{3r}} \right\rfloor$	$\left\lfloor \frac{N_\mathrm{g}\sqrt{r}}{\sqrt{3}} \right\rfloor$
p31m (3*3)	3	6	$2\pi/3$	1	$\left\lfloor \frac{N_\mathrm{g}}{\sqrt{3r}} \right\rfloor$	$\left\lfloor \frac{N_\mathrm{g}\sqrt{r}}{\sqrt{3}} \right\rfloor$
p4g (4*2)	3	8	$\pi/2$	1	$\left\lfloor \frac{N_\mathrm{g}}{2\sqrt{r}} \right\rfloor$	$\left\lfloor \frac{N_\mathrm{g}\sqrt{r}}{2} \right\rfloor$
cmm (2*22)	3	4	$\pi/2$	$\{1, \sqrt{2}, \sqrt{3}, 2\}$	$\left\lfloor \frac{N_\mathrm{g}}{\sqrt{2}} \right\rfloor$	$\left\lfloor \frac{N_\mathrm{g}}{\sqrt{2}} \right\rfloor$
pmg (22*)	4	4	$\pi/2$	$\{1, \sqrt{2}, \sqrt{3}, 2\}$	$\left\lfloor \frac{N_\mathrm{g}}{2\sqrt{r}} \right\rfloor$	$\left\lfloor \frac{N_\mathrm{g}\sqrt{r}}{2} \right\rfloor$
pg (xx)	4	2	$\pi/2$	$\{1, \sqrt{2}, \sqrt{3}, 2\}$	$\left\lfloor \frac{N_\mathrm{g}}{\sqrt{2r}} \right\rfloor$	$\left\lfloor \frac{N_\mathrm{g}\sqrt{r}}{\sqrt{2}} \right\rfloor$
cm (*x)	4	2	$\pi/2$	$\{1, \sqrt{2}, \sqrt{3}, 2\}$	$\left\lfloor \frac{N_\mathrm{g}}{\sqrt{2r}} \right\rfloor$	$\left\lfloor \frac{N_\mathrm{g}\sqrt{r}}{\sqrt{2}} \right\rfloor$
pgg (22x)	4	4	$\pi/2$	$\{1, \sqrt{2}, \sqrt{3}, 2\}$	$\left\lfloor \frac{N_\mathrm{g}}{2\sqrt{r}} \right\rfloor$	$\left\lfloor \frac{N_\mathrm{g}\sqrt{r}}{2} \right\rfloor$

Supplementary Table II: Geometry of different fundamental domains explored for each group. For each wallpaper group except p1 (o) we list the number of sides the fundamental domain has, N_s, the number of these domains per primitive cell, N_d, the angle, α, length ratio, r, and the number of nodes along each side, N_1 and N_2, corresponding to Supplementary Tab. I.

SUPPLEMENTARY METHODS

To make an approximate comparison between the total number of configurations that can be generated combinatorially on a p1 cell and the total number which can be generated by using the symmetry of the remaining 16 groups in a similar area, the total number of nodes in the p1 group's primitive cell is used to estimate the node edge-length density (number of nodes per unit length) needed in each of the other wallpaper groups. In principle, we would like to describe the same area of two-dimensional space outlined in cyan on the left in Supplementary Fig. 9 as that on the right, but now subject to new symmetry constraints which reduce the degrees of freedom within that area. For the p1 group, the primitive cell itself is the fundamental domain, whereas in the other groups, two or more fundamental domains exist within their primitive cell; therefore the number of nodes per fundamental domain will be lower in other groups compared to p1.

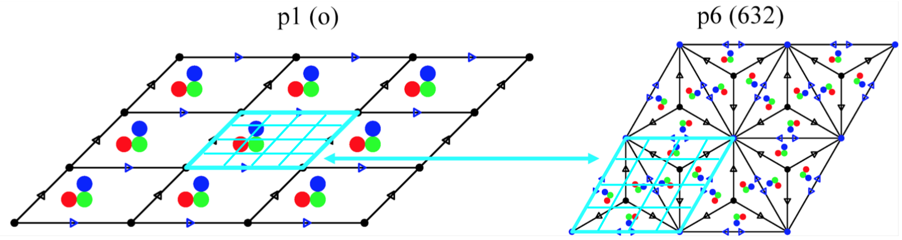

Supplementary Figure 9: Comparing the primitive cells of p1 and p6.

As a basis, consider a p1 primitive cell with N_{g}^2 total nodes distributed over the cell. Each group's primitive cell (depicted in cyan in Supplementary Tab. I) can then be used to compute the number of nodes along the edges of its fundamental domain needed to achieve the same overall number of nodes in its primitive cell. Whenever possible, we try to place nodes with the same edge-length density on both sides of the fundamental domain; to this end, we define the ratio of the lengths of the sides of the parallelogram defining a group's fundamental domain as $r = \frac{L_2}{L_1} = \frac{N_2}{N_1} \geq 1$, where N_i denotes the number of nodes along edge i. In general, wallpaper groups admit the possibility of $r \neq 1$, though it is forbidden for certain groups (*cf.* Supplementary Tab. I); there are two notable exceptions where $\frac{L_2}{L_1} \neq \frac{N_2}{N_1}$ which we will address later. Regardless, where r is variable we sample a discrete set of different r values. Setting the node edge-length density of the p1 primitive cell to that of a chosen group yields:

$$\frac{N_{\rm g}^2}{A_{\rm g}N_{\rm d}} = \frac{N_1 N_2 \left(1 - \frac{1}{2} \times (N_{\rm s} \bmod 2)\right)}{A_{\rm g}}, \tag{1}$$

where $A_{\rm g}$ is the area of a fundamental domain for a given group, $N_{\rm s}$ is the number of sides the domain has, and $N_{\rm d}$ is the number of fundamental domains per primitive cell. The modulo operation exists to account for the fact that triangular domains are only half the size of a parallelogram and so encompass only half of the $N_1 \times N_2$ grid. Incorporating the fact that $N_2 = rN_1$, then we arrive at:

$$N_1 = \left\lfloor \sqrt{\frac{N_{\rm g}^2}{rN_{\rm d}\left(1 - \frac{1}{2} \times (N_{\rm s} \bmod 2)\right)}} \right\rfloor, \tag{2}$$

where we have rounded the number down to the nearest integer. Rounding down to the nearest integer prevents the congruent primitive cell of the other group from exceeding the number of nodes in the p1 cell; N_1 could also be rounded up or to the nearest integer, however, this does not qualitatively affect the conclusions reached in this work. It follows that $N_2 = \lfloor rN_1 \rfloor$.

We have covered the fundamental domains with a regular grid such that nodes lie along the diagonal of a parallelogram (*cf.* Supplementary Fig. 1) when the fundamental domain is triangular (formed by dividing the parallelogram in half). In our convention, when a fundamental domain is triangular and $r \neq 1$ the edge density of nodes on each side of the domain is necessarily unequal and prevents us from placing nodes along the edges with the same spacing. To maintain that the diagonal of the parallelogram intersects nodes in these cases, we instead set $N_2 = N_1$ even when $L_2 \neq L_1$, thus r does not affect the value of N_2. This occurs in two instances: cmm and p6m; consequently, the node density of these wallpaper groups is independent of r in Supplementary Tab. II. In simulations to compute phase diagrams we considered all of the r and α values listed in the Supplementary Tab. II for each group.

Under certain circumstances our algorithm can produce identical structures using different prescriptions from different wallpaper groups. The cause of this can be two-fold: first, a prescription may not call for placing any colloids along an edge (or edges) that distinguish two groups from each other which are otherwise similar, and thus, the two resulting configurations do not exploit the symmetry constraints that otherwise differentiate the two groups; second, there may not be enough colloids placed to distinguish the chirality, or rel-

ative orientation of fundamental domains between groups that may be a result of different operations. As an example of the second condition, consider the case where a single colloid is placed in the center of a fundamental domain's face. A reflection of this face would look identical to a translation.

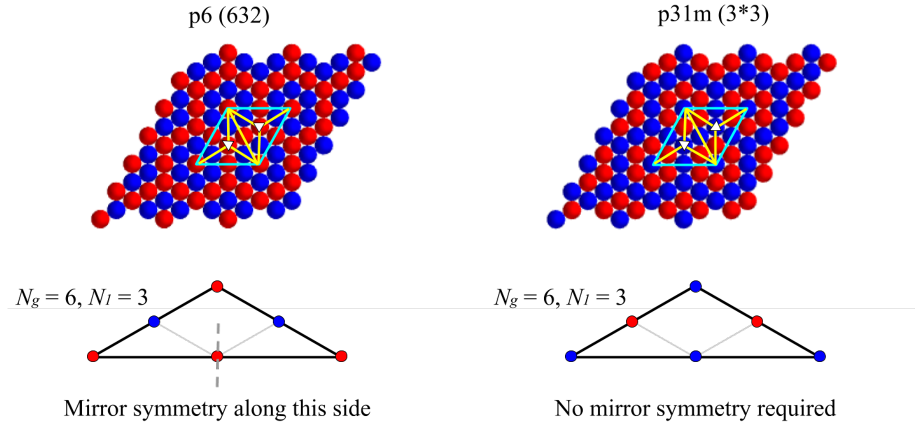

Supplementary Figure 10: The primitive cell for both the p6 and p31m groups is superimposed in cyan over their respective structures. The fundamental domain boundaries inside the primitive cell are drawn in yellow, and reproduced below the lattice in black. There are two centers of rotation drawn in each primitive cell, denoted by the white triangles. Their relative directions are indicated by the direction of triangles (*cf.* Supplementary Tab. I). In p6, the centers rotate in the same direction imposing a mirror along the longest side of the fundamental domain, whereas in p31m the centers rotate in the opposite direction and do not impose this condition.

Consider the two configurations resulting from the p6 and p31m groups as reported in Fig. 2 of the main text, reproduced in Supplementary Fig. 10. The structures are the same, though the labeling (red vs. blue) is opposite, which is arbitrary. To see how this can result, we have superimposed each group's primitive cell (cyan) and fundamental domains (yellow). In this case, we specified $N_g = 6$ which results in $N_1 = 3$ for both groups. The fundamental domains for both groups are isosceles triangles where $\alpha = 2\pi/3$ (*cf.* Supplementary Tab. I) and have two centers of rotation inside their primitive cells. Here, we sought a 1:1 stoichiometry and the solution represented is the one where all lattice sites are filled. In the p6 wallpaper group, the three fundamental domains rotate in the same direction about each rotation center, whereas in the p31m group, they rotate in opposite

directions. The ultimate consequence is that the p6 group requires mirror symmetry along its fundamental domain's longest edge which is not required by p31m. However, in the case of $N_1 = 3$ there are not enough sites to cause this distinguishing feature to manifest. Using $N_g = 8$ would result in $N_1 = 4$ which would permit this to appear; this is partly why we used $N_g = 8$ in addition to $N_g = 6$ in this work.

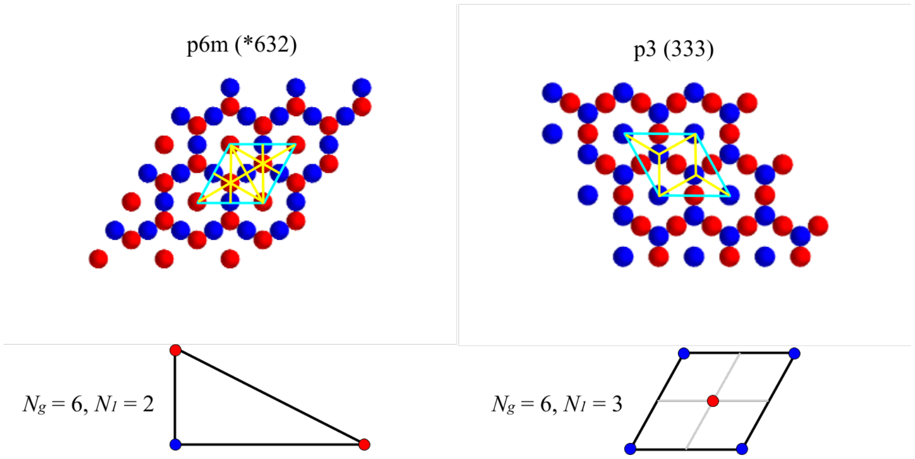

Supplementary Figure 11: The primitive cell for both the p6m and p3 groups is superimposed in cyan over their respective structures. The fundamental domain boundaries inside the primitive cell are drawn in yellow, and reproduced below the lattice in black. For $N_g = 6$, the p6m group has $N_1 = 2$ permitting only a single solution to achieve a 1:1 stoichiometric ratio, whereas the p3 group has $N_1 = 3$ and permits several different solutions. Only the solution of interest for the latter is depicted.

As a second example, consider the p3 and p6m groups as reported in Fig. 2 of the main text. Again, the same structure, albeit with opposite coloring, for $N_g = 6$ with a 1:1 stoichiometry can result; these lattices are reproduced in Supplementary Fig. 11. In this case, N_1 is different for the two groups because p6m has 12 fundamental domains per primitive cell, while p3 has only 3. In this case the p6m group offers only a single solution to achieving a 1:1 stoichiometric ratio. There are multiple ways to do this for p3, however, the solution chosen makes use of only a single face site and all corners, neglecting the edges entirely. The consequence is that two identical configurations can be found originating in different groups.

A single, representative basin hopping[1–3] result is given in Fig. 3 of the main text. Here we illustrate additional examples for a binary mixture. In each case that follows, a 1:1

stoichiometry was used. We set $N_{\mathrm{g}} = 6$ and exhaustively enumerated all possible primitive cell configurations for all 16 groups considered. These configurations were scaled to contact such that the nearest two colloids were $r_{\mathrm{min}} = 2^{1/6}\sigma$ apart; the potential energy per total number of colloids was subsequently evaluated and ranked from lowest to highest. In all cases listed here, the ground state (lowest energy configuration) was found in this initial list. To ascertain this, we further optimized up to 25 structures with the lowest energy from each of the 16 wallpaper groups, besides p1. In all examples which follow, the ranked energies of these unrelaxed initial candidates are shown in the inset; the plateau at the lowest energy shown demonstrates multiple discoveries of the (same) ground state in each instance. The basin hopping optimization step was performed with $\hat{T} = 0.5$ and run for 1500 iterations. Each iteration involved the perturbation of a randomly chosen set of atom coordinates, and potentially, the primitive cell's shape and size. The coordinates and box shape were then deterministically minimized with the L-BFGS-B algorithm[4] to produce a new candidate configuration which was accepted with a probability of $p_{\mathrm{acc}} = \min\left[1, \left(-\frac{\Delta(U/N_{\mathrm{tot}})}{\hat{T}}\right)\right]$, where U is the potential energy, N_{tot} is the total number of colloids in the primitive cell, and the Δ refers to the difference between the final and initial states (*cf.* main text). Following basin hopping, the lowest energy configuration found during this stage was deterministically optimized with the Nelder-Mead simplex method.[5] The radial distribution functions were compared to identify the structurally unique candidates with the lowest energies as described in the main text. Various unique, representative candidates are depicted at characteristic locations along the sorted energy landscape. In the main text, we generally report the results from $N_{\mathrm{g}} = 8$ from which we drew configurations randomly to be optimized with basin hopping. The exhaustive approach with $N_{\mathrm{g}} = 6$ and this stochastic one with $N_{\mathrm{g}} = 8$ yielded the same final result for the ground state in all cases.

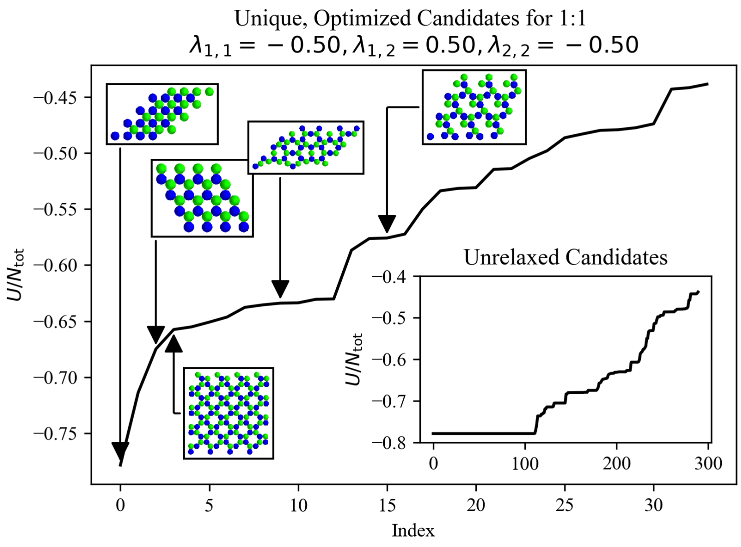

Supplementary Figure 12: Basin hopping optimization of a binary mixture at $x_1 = 0.5$ for $\lambda_{1,1} = -0.50, \lambda_{1,2} = 0.50, \lambda_{2,2} = -0.50$. Species 1 is depicted in blue, species 2 in green. The ground state corresponds to the leftmost structure. The inset depicts the (sorted) energy of all candidates that were chosen to be optimized; only the structurally unique results of those optimizations are reported in the main panel, with certain representative structures shown along the energy landscape.

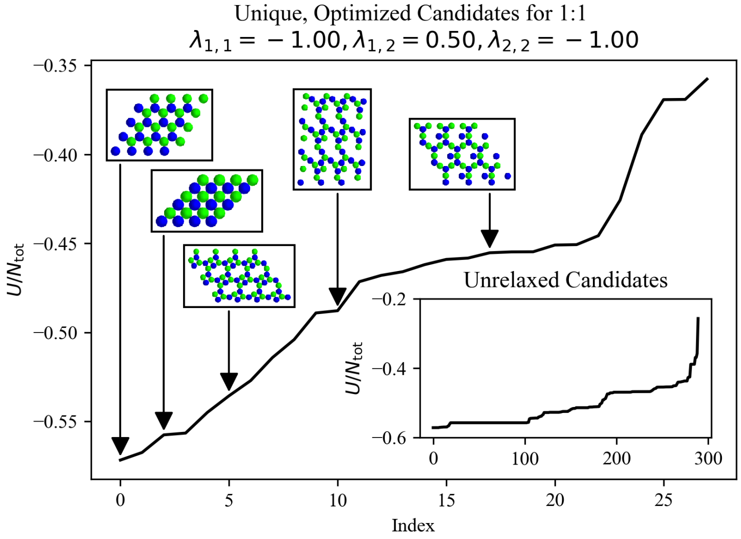

Supplementary Figure 13: Basin hopping optimization of a binary mixture at $x_1 = 0.5$ for $\lambda_{1,1} = -1.00, \lambda_{1,2} = 0.50, \lambda_{2,2} = -1.00$. Species 1 is depicted in blue, species 2 in green. The ground state corresponds to the leftmost structure. The inset depicts the (sorted) energy of all candidates that were chosen to be optimized; only the structurally unique results of those optimizations are reported in the main panel, with certain representative structures shown along the energy landscape.

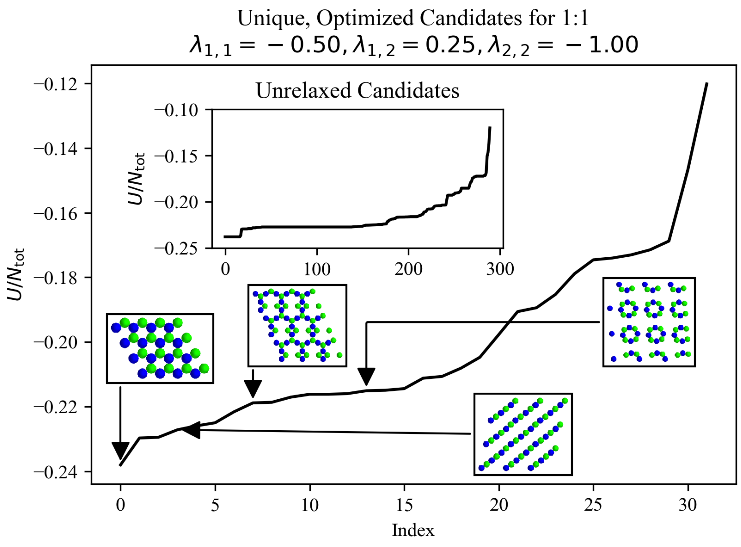

Supplementary Figure 14: Basin hopping optimization of a binary mixture at $x_1 = 0.5$ for $\lambda_{1,1} = -0.50, \lambda_{1,2} = 0.25, \lambda_{2,2} = -1.00$. Species 1 is depicted in blue, species 2 in green. The ground state corresponds to the leftmost structure. The inset depicts the (sorted) energy of all candidates that were chosen to be optimized; only the structurally unique results of those optimizations are reported in the main panel, with certain representative structures shown along the energy landscape.

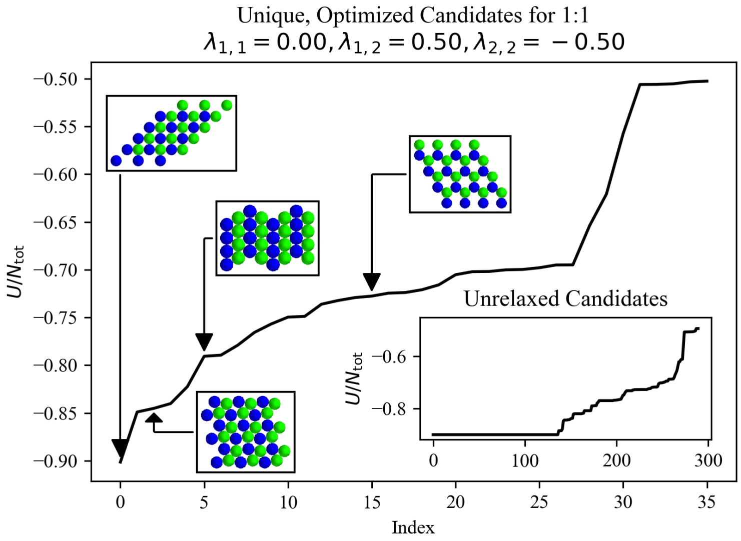

Supplementary Figure 15: Basin hopping optimization of a binary mixture at $x_1 = 0.5$ for $\lambda_{1,1} = 0.00, \lambda_{1,2} = 0.50, \lambda_{2,2} = -0.50$. Species 1 is depicted in blue, species 2 in green. The ground state corresponds to the leftmost structure. The inset depicts the (sorted) energy of all candidates that were chosen to be optimized; only the structurally unique results of those optimizations are reported in the main panel, with certain representative structures shown along the energy landscape.

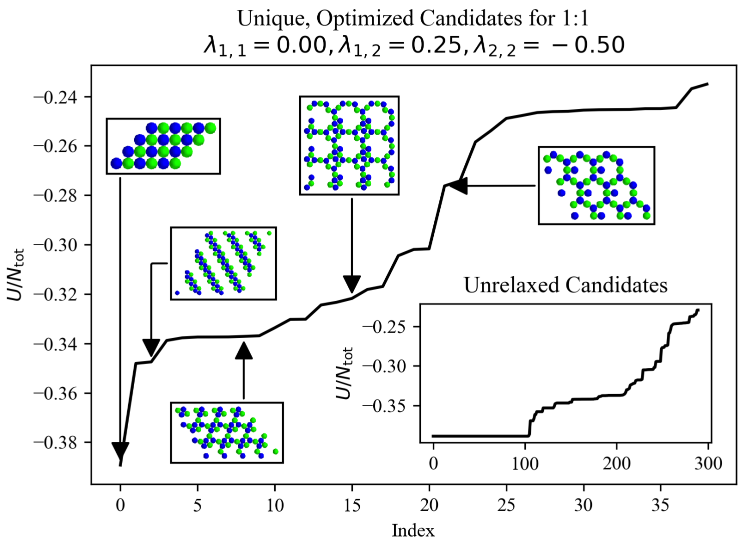

Supplementary Figure 16: Basin hopping optimization of a binary mixture at $x_1 = 0.5$ for $\lambda_{1,1} = 0.00, \lambda_{1,2} = 0.25, \lambda_{2,2} = -0.50$. Species 1 is depicted in blue, species 2 in green. The ground state corresponds to the leftmost structure. The inset depicts the (sorted) energy of all candidates that were chosen to be optimized; only the structurally unique results of those optimizations are reported in the main panel, with certain representative structures shown along the energy landscape.

SUPPLEMENTARY NOTE 1

For $N_{\mathrm{g}} = 6$ all solutions to some of the most numerous groups in the binary case with a 1:1 stoichiometry were evaluated when scaled to contact. The best fit of their energy histograms to a normal distribution is drawn in orange for several different sets of $\boldsymbol{\lambda}$ values in Supplementary Fig. 17.

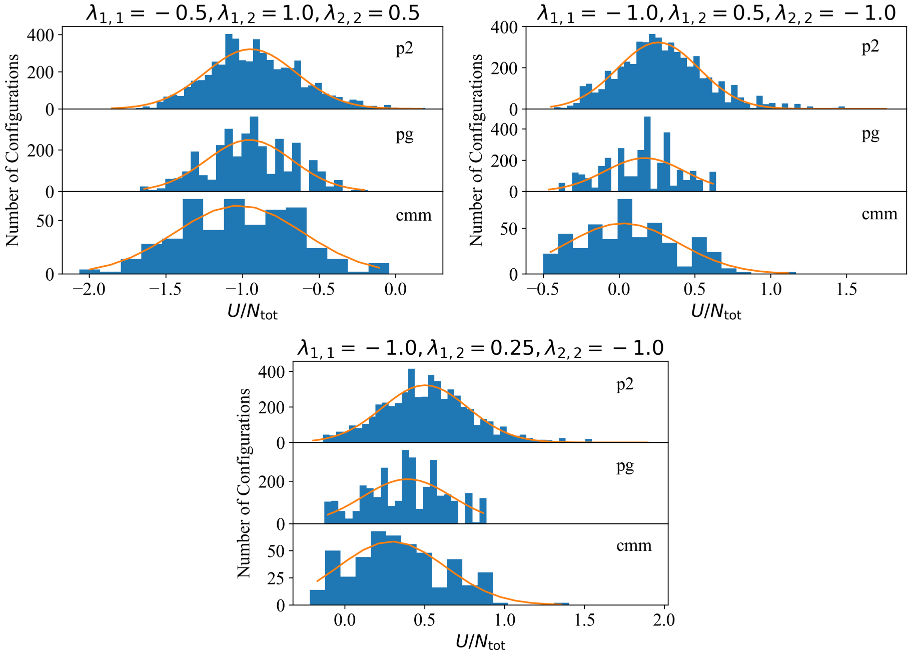

Supplementary Figure 17: Potential energy distribution of all lattices satisfying a 1:1 stoichiometry for several groups with various different sets of pairwise potentials.

SUPPLEMENTARY DISCUSSION

The equatorial regions of three quadrants in Fig. 5 in the main text (shades of green) are dominated by hexagonal arrays of particles in coexistence with either other hexagonal arrays or gas-like phases. Here species' self-interactions dominate, and at least one is strongly attractive relative to their cross-interactions which prevents the components from mixing. Moving to the zenith increases the cross-interaction, which is always favorable in this hemisphere, leading to mixed lattices with 6-fold rotation symmetry (if species identity is neglected). In the case of $x_1 = 0.5$ (1:1 stoichiometry) lattices with alternating layers are found, which have the same stoichiometry. As the stoichiometry is increased, 6-fold lattices tend to form which are identical to the stoichiometry of the bulk solution. For $x_1 = 0.66$ (2:1 stoichiometry) a honeycomb lattice forms, while for $x_1 = 0.75$ (3:1 stoichiometry) Kagome lattices tend to dominate. This is not entirely universal, and clearly lattices with stoichiometries that are different the bulk form in certain regions of these quadrants as well; for example, a small region of alternating layer structures persists even up to $x_1 = 0.75$.

The lower left quadrant, however, reflects a much greater diversity of structures. In this region, the self-interaction parameters are negative, reflecting a repulsive tendency between species of the same type; such repulsion is mitigated by the favorable cross-interaction between unlike components. This reflects the general scenario found in DNA-based assembly; however, only the $\lambda_{1,1} = \lambda_{2,2}$ plane (a line in the projection of Fig. 5) may be accessed without the use of multi-flavoring. For the case of $x_1 = 0.5$, Fig. 5 is symmetric about this line since the identities of the two particles can simply be exchanged to transform the results from one half of the diagram to the other. Starting from the equator and moving to the zenith corresponds to increasing the cohesion between different species and results in a systematic increase in the rotational symmetry of the resulting lattice. Along the equator the system begins in a disordered binary gas state ($\lambda_{1,2} = 0$); this is followed by a progression through alternating strings (2-fold symmetry), an open honeycomb lattice (3-fold), and a square lattice (4-fold) before reaching the zenith where the alternating layer (6-fold) is the most stable morphology.

The ternary phase diagram presented in the main text only includes certain landmark structures. A complete diagram showing all the structures found on the hull is given in Supplementary Fig. 18. As a reference, in Supplementary Fig. 19 we also depict the coor-

dinates that are sampled if we chose to investigate systems with stoichiometries containing up to some maximum; *i.e.*, if a structure has a stoichiometry of ξ_1:ξ_2:ξ_3, the maximum stoichiometry refers to $\max[\xi_i]$.

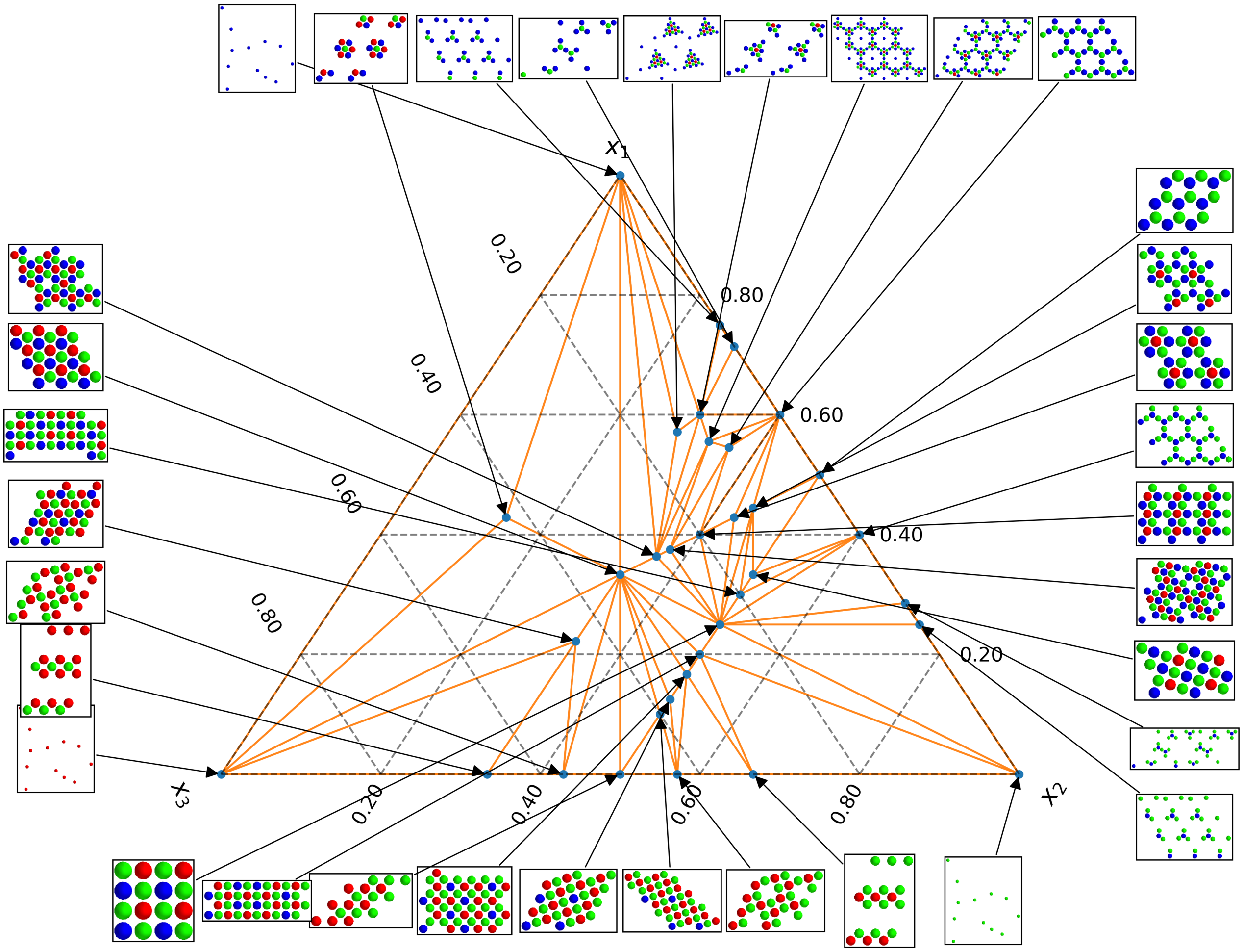

Supplementary Figure 18: Complete ternary ground-state phase diagram with all thermodynamically stable structures indicated.

Beginning from ($x_1 = 1/2, x_2 = 1/2, x_3 = 0$) and moving along the 4-fold line (blue arrow in Fig. 6), we see that the system essentially transforms into a variety of square lattices that depend on the system's composition. From the same 3-fold starting point, when moving along the 6-fold line (green arrow in main text) the system undergoes an intuitive transformation in which the vacancies present in the open honeycomb lattice composed of species 1 and 2 are continuously filled by species 3. The fully filled lattice is shown at

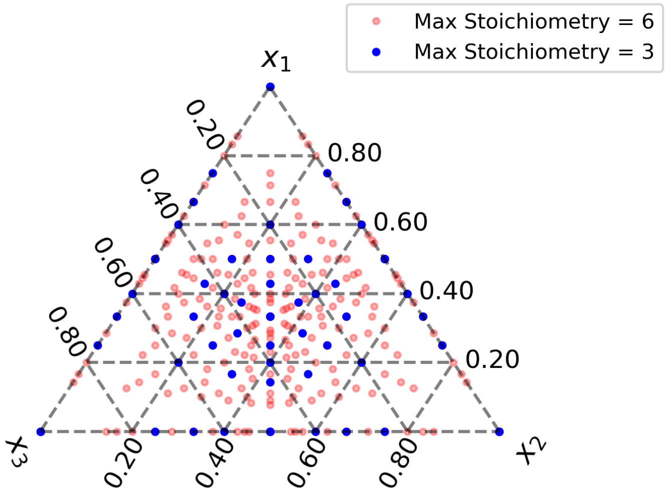

Supplementary Figure 19: Coordinates sampled when the maximum stoichiometry is 3 compared to when it is 6.

($x_1 = x_2 = x_3 = 1/3$). Once all the vacancies are filled, adding more of species 3 forces the system to undergo a phase transition into structures with different characteristics, which depend on the composition of the system. Generally, these include various 6-fold and 4-fold lattices, and even cluster phases.

We validated our predictions of the ground state ternary phase diagram presented in the main text by performing molecular dynamics simulations. For these simulations the total number of particles was $N_{\text{tot}} = 2000$, the density was set to $\rho^* = 0.10$, and a range of temperatures, $T^* = 0.02, 0.03, \ldots, 0.06$, was explored which gave consistent results. Representative snapshots for a single condition are given in Supplementary Figs. 20 and 21 along with the predicted coexisting phases above each snapshot. For these systems, $\lambda_{1,1} = -1.0, \lambda_{1,2} = 0.5, \lambda_{1,3} = 0.0, \lambda_{2,2} = -1.0, \lambda_{2,3} = 1.0, \lambda_{3,3} = -1.0$. Species 1 is depicted in blue, species 2 in green, and species 3 in red.

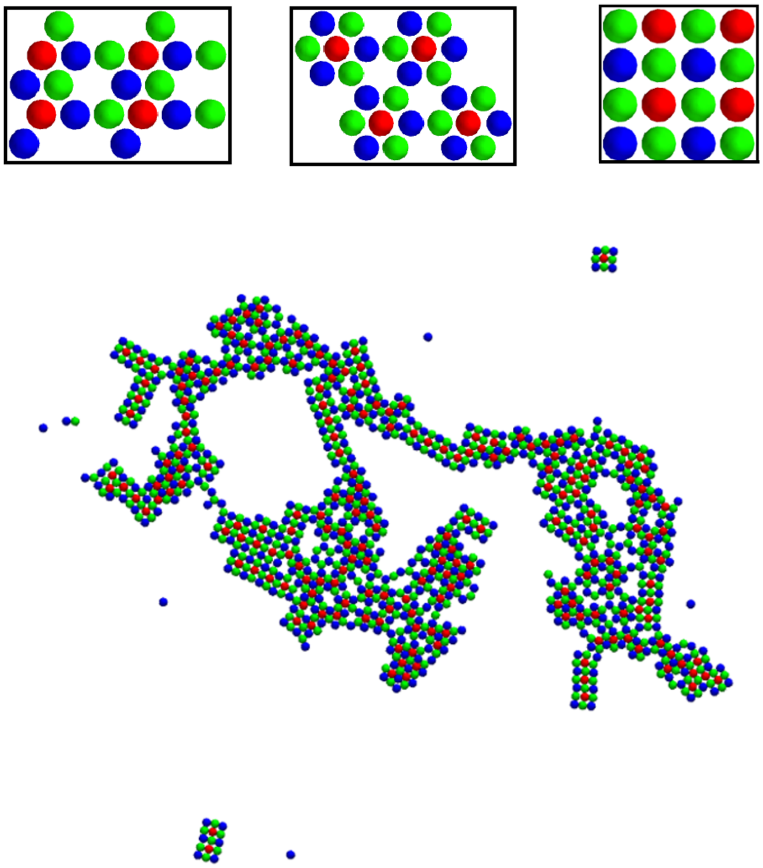

Supplementary Figure 20: Molecular dynamics snapshot from a system at $x_1 = 0.40, x_2 = 0.42, x_3 = 0.18$, $T^* = 0.04$. The structures which are predicted to be stable at these conditions are shown above in black boxes.

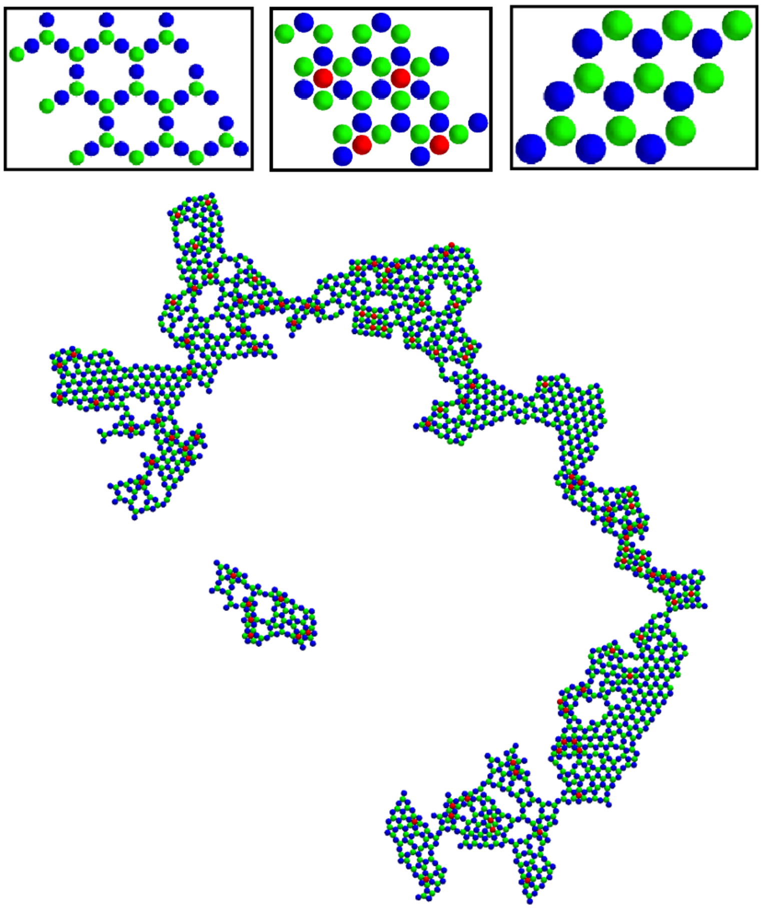

Supplementary Figure 21: Molecular dynamics snapshot from a system at $x_1 = 0.50, x_2 = 0.45, x_3 = 0.05$, $T^* = 0.02$. The structures which are predicted to be stable at these conditions are shown above in black boxes.

SUPPLEMENTARY REFERENCES

[1] D. J. Wales and J. P. K. Doye, "Global optimization by basin-hopping and the lowest energy structures of lennard-jones clusters containing up to 110 atoms," Journal of Physical Chemistry A **101**, 5111–5116 (1997).

[2] D. J. Wales and H. A. Scheraga, "Global optimization of clusters, crystals, and biomolecules," Science **285**, 1368—1372 (1999).

[3] E. Jones, T. Oliphant, P. Peterson, and et al., "SciPy: Open source scientific tools for Python," http://www.scipy.org/ (2001).

[4] R. H. Byrd, L. P., J. Nocedal, and C. Zhu, "A limited memory algorithm for bound constrained optimization," SIAM Journal on Scientific Computing **16**, 1190–1208 (1995).

[5] J. A. Nelder and R. Meade, "A simplex method for function minimization," The Computer Journal **7**, 308–313 (1965).
